# Humoral and Cell-Mediated Immunity to Pandemic H1N1 Influenza in a Canadian Cohort One Year Post-Pandemic: Implications for Vaccination

**DOI:** 10.1371/journal.pone.0028063

**Published:** 2011-11-23

**Authors:** Lisa E. Wagar, Laura Rosella, Natasha Crowcroft, Beth Lowcock, Paulina C. Drohomyrecky, Julie Foisy, Jonathan Gubbay, Anu Rebbapragada, Anne-Luise Winter, Camille Achonu, Brian J. Ward, Tania H. Watts

**Affiliations:** 1 Department of Immunology, University of Toronto, Toronto, Ontario, Canada; 2 Public Health Ontario, Toronto, Ontario, Canada; 3 Dalla Lana School of Public Health, University of Toronto, Toronto, Ontario, Canada; 4 Department of Laboratory Medicine and Pathobiology, University of Toronto, Toronto, Ontario, Canada; 5 Research Institute of the McGill University Health Centre, Montreal, Quebec, Canada; University of Georgia, United States of America

## Abstract

We evaluated a cohort of Canadian donors for T cell and antibody responses against influenza A/California/7/2009 (pH1N1) at 8-10 months after the 2nd pandemic wave by flow cytometry and microneutralization assays. Memory CD8 T cell responses to pH1N1 were detectable in 58% (61/105) of donors. These responses were largely due to cross-reactive CD8 T cell epitopes as, for those donors tested, similar recall responses were obtained to A/California 2009 and A/PR8 1934 H1N1 Hviruses. Longitudinal analysis of a single infected individual showed only a small and transient increase in neutralizing antibody levels, but a robust CD8 T cell response that rose rapidly post symptom onset, peaking at 3 weeks, followed by a gradual decline to the baseline levels seen in a seroprevalence cohort post-pandemic. The magnitude of the influenza-specific CD8 T cell memory response at one year post-pandemic was similar in cases and controls as well as in vaccinated and unvaccinated donors, suggesting that any T cell boosting from infection was transient. Pandemic H1-specific antibodies were only detectable in approximately half of vaccinated donors. However, those who were vaccinated within a few months following infection had the highest persisting antibody titers, suggesting that vaccination shortly after influenza infection can boost or sustain antibody levels. For the most part the circulating influenza-specific T cell and serum antibody levels in the population at one year post-pandemic were not different between cases and controls, suggesting that natural infection does not lead to higher long term T cell and antibody responses in donors with pre-existing immunity to influenza. However, based on the responses of one longitudinal donor, it is possible for a small population of pre-existing cross-reactive memory CD8 T cells to expand rapidly following infection and this response may aid in viral clearance and contribute to a lessening of disease severity.

## Introduction

A novel swine-origin H1N1 influenza virus (pH1N1) emerged in North America in mid-April of 2009, resulting in widespread infection [1], [2]. The infectious behavior of the novel 2009 strain met pandemic criteria set by the World Health Organization in mid-June, 2009. A second wave of infection with the same strain occurred in the autumn of 2009. By August 2010, influenza outbreaks had subsided and influenza incidence in the population had returned to normal seasonal rates. Contrary to typical seasonal influenza, attack rates were observed to be highest in younger people [1], [3], [4]. However, infection in older age groups resulted in more severe illness and increased mortality rates compared to the general population [3], [5], [6]. It has been suggested that older people who had been exposed to an H1N1 influenza from the early 20^th^ century may have been protected by pre-existing cross-reactive antibodies [7], [8], as strains originating from the 1918 pandemic are antigenically similar to the 2009 strain [9]. T cells produced against pH1N1 2009 are able to respond to challenge with the 1918 pandemic H1N1 strain [10] and memory T cells generated against past seasonal infections can respond to pH1N1 challenge [11]–[13], suggesting that T cell cross-reactivity exists in primed hosts.

While it has been established that influenza-specific B cell memory can be very long-lived [8], [14], there are limited data on the magnitude and persistence of antibody and T cell responses to influenza post-pandemic. To address this, we analyzed humoral and T cell-mediated immunity to pH1N1 in a cross-sectional cohort of the Toronto population, approximately 8-10 months post 2009 pandemic as well as before, during and after infection of one donor from whom a series of longitudinal samples was available.

## Materials and Methods

### Ethics statement

Ethics approval was granted by the Research Ethics Board of the University of Toronto. All subjects gave written informed consent.

### Study design and sample collection

Individuals who were at least 18 years of age were invited to participate in a case/control or a seroprevalence cohort study. Individuals self-reported vaccination in all study groups. The vaccine they would have received through the publicly funded Canadian vaccine program was the GlaxoSmithKline monovalent, inactivated, split-virion pandemic H1N1 influenza vaccine containing 3.75 µg hemagglutinin (HA) with AS03 adjuvant (unadjuvanted vaccine was also available but was only given to pregnant women and young children). Donors reported vaccination with the pandemic H1N1 vaccine from October 2009 to January 2010.

#### Case/control cohort

Case/control donors (the Ontario population of a previous study [15]) were recruited during early autumn of 2009. All participants had medically attended influenza-like illness (ILI) and were subsequently tested for influenza A/California7/2009-like strains by PCR using nasopharyngeal swabs, performed from April to November 2009, largely prior to vaccine availability. Case/control volunteers provided blood for influenza-specific antibody and T cell testing in July-August of 2010, approximately 8–10 months after initial PCR testing for pH1N1. Case participant ages ranged from 19–76, with a mean age of 44; control participants were aged 29–74, with a mean age of 51.

#### Seroprevalence cohort

A seroprevalence study was undertaken beginning August 2009 [16]; Toronto residents were recruited through an advertising/email/web-based campaign; those who completed an online questionnaire were invited to give a blood sample. From April-June 2010, participants were asked to provide a second blood sample and complete a questionnaire on risk factors, health status (such as ILI) and vaccination history. The mean participant age for this cohort was 50, with an age range of 24-76.

#### Longitudinal analysis

Longitudinal samples were available from one subject (who was not part of the case/control or seroprevalence studies), a 55-year old male with no co-morbid conditions, allergies, or relevant medications who reported an influenza-like illness in mid-June 2009 and was confirmed PCR positive for pH1N1. He exhibited general malaise with a modest fever, which began 4–5 days after onset of illness and a mild-moderate dry cough that began approximately one week post-onset. Peripheral blood mononuclear cells (PBMC) had been stored for this individual over one year prior to the onset of symptoms and further samples were obtained at several times during and after infection. This donor was subsequently vaccinated (through the publically available Canadian vaccine program) for influenza approximately 20 weeks (GSK AS03-adjuvanted monovalent vaccine) and 17 months (seasonal trivalent inactivated vaccine, including A/California/07/2009) post-infection with pH1N1.

### Viruses and media

A/California/07/2009-like virus (H1N1) was isolated from a nasopharangeal swab by Public Health Ontario laboratories and confirmed at the National Microbiology Laboratory using a hemagglutination inhibition (HAI) assay. Influenza A/Puerto Rico/8/34 (PR8) was grown in embryonated chicken eggs. Dr. Pamela S. Ohashi, University Health Network, Toronto, kindly provided the Lymphocytic choriomeningitis virus (LCMV) Armstrong. Complete PBMC medium was RPMI 1640 HEPES modification (Sigma-Aldrich), 10% fetal bovine serum (FBS, Invitrogen), 1% non-essential amino acids (Invitrogen), 100 U/mL penicillin, 0.1mg/mL streptomycin, 1 mM L-glutamine, 1 mM sodium pyruvate, and 0.1% β-mercaptoethanol.

### pH1N1-specific antibody detection in human serum

Serum antibodies were detected by microneutralization (MN), adapted from the World Health Organization method [17] or HAI, adapted from the World Health Organization method [18]. An ELISA similar to that recently reported by Mavrouli and colleagues [19] was optimized to detect antibodies binding to recombinant pandemic H1 hemagglutinin. Briefly, wells of enzyme immunoassay/RIA flat-bottom 96-well plates (Costar) were coated with 0.4 µg/well recombinant HA derived from strains H1N1 A/California/07/2009 or A/California/04/2009, H1N1 A/Brisbane/59/2007, H3N2 A/Brisbane/10/2007, or H9N2 A/Hong Kong/1073/1999 (all from Protein Sciences Corporation) and blocked with 1% bovine serum albumin. Human sera were plated in serial two-fold dilutions and incubated for 2 h at room temperature. HRP-conjugated anti-human IgG (Sigma, St. Louis, MO) diluted 1/6000 was added for 1 h at room temperature followed by addition of the substrate for visualization. Plates were read with a Multiskan EX spectrometer (Thermo Electron) at 405 nm. Optical density of samples was corrected for background based on the OD of uncoated, serum-treated wells. Titers reported herein were determined by microneutralization unless otherwise noted.

### Assessment of T cell responses to live influenza virus challenge

PBMC were isolated from whole blood by Ficoll density gradient separation and frozen in FBS with 20% DMSO (Sigma). Cryopreserved PBMC were thawed in complete medium, plated at 1 million cells per well in a 96-well round-bottom microtitre plate and infected with 1 hemagglutination unit (HAU) of A/California/7/2009, 5 HAU of PR8, or 2000 PFU of LCMV Armstrong per well and incubated for 18 hours at 37°C, 5% CO_2_. Brefeldin A (BD Biosciences) was added for the final 6 hours of stimulation at 1.5 µL/mL.

### Flow cytometry

Anti-human monoclonal antibodies used were CD27, CD8, CD3, IFNγ, CD45RA, and TNFα (eBioscience), granzyme B (Invitrogen), and CD107a (BD Biosciences). Anti-CD107a was added for the final 6 hours of stimulation. For detecting intracellular cytokines, PBMC were surface stained, fixed and permeabilized using Cytokfix/Cytoperm buffers (BD Biosciences), washed, and stained intracellularly for 30 minutes at 4°C. Data were collected using an LSR II (BD Biosciences). Background fluorescence was established with fluorescence minus one (FMO) controls except CD107a, for which an isotype control was used. Approximately 1×10^5^ to 6.5×10^5^ total T cell events were collected per condition per donor.

### Data Analysis

FlowJo (TreeStar) was used to gate and analyze flow cytometry data. Plotting and statistical analyses were performed with Prism (GraphPad) software.

## Results

### Whole virus stimulation assay to detect T cell responses to pH1N1

**Figure 1:**
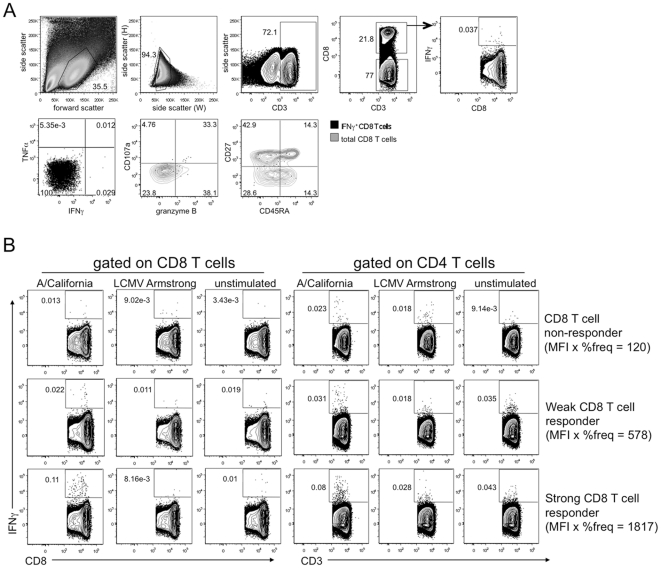
Detection of influenza-responsive CD8 T cells by multicolour flow cytometry. Total PBMC were stimulated for 18 hours with pH1N1 influenza, or as a control, with LCMV Armstrong, or left unstimulated and then assessed for IFNγ production by intracellular cytokine staining and flow cytometry. Gates are based on fluorescence minus one controls. (A) Representative gating used to identify IFNγ^+^ CD8 T cells from total PBMC. (B) Sample non-responder, weak responder, and strong responder to pH1N1 identified in the Toronto cohort 8-10 months post-pandemic; positive versus non-responder is defined in the results. A representative “weak” responder was arbitrarily chosen from the bottom third of positive responses whereas the “strong” responder was from the top third of responders.

To identify influenza-specific memory T cells in a Toronto population post-pandemic, we used flow cytometry analysis following whole virus stimulation of PBMC. Flow cytometry was chosen over ELiSPOT as it offered the advantage of multi-parameter analysis. Influenza-responsive CD8 and CD4 T cells were identified on the basis of their production of IFNγ, after 18hrs stimulation (Figure 1A). As 18h is too short a time frame for the cells to proliferate, this restimulation assay reports the frequency of influenza-specific memory T cells circulating in the individual at the time of PBMC harvest. To rule out non-specific innate immune responses to viral stimulation ex vivo, we stimulated PBMC with a rodent virus LCMV to which we did not expect recall responses and for the most part, the results were similar to those obtained with unstimulated controls (Figure 1B). By analyzing the product of the median fluorescence intensity (MFI) and the frequency of T cells with IFNγ staining, the response value reported takes into account both the proportion of cells responding to influenza virus and the amount of IFNγ per cell. We defined a cutoff for CD8 T cell responses based on these parameters. A donor's response to influenza was considered positive if: a) the frequency of IFNγ^+^ CD8 T cells was greater than that of stimulation controls, b) the frequency of IFNγ^+^ cells was greater than 0.01% of the total CD8 T cell pool, and c) the product of the MFI and the frequency of IFNγ^+^ CD8 T cells was at least two-fold above the control cultures' values. IFNγ staining of T cell populations for a representative CD8 T cell pH1N1 non-responder, weak responder, and strong responder is shown in Figure 1B and a summary of results for each cohort is described in Table 1.

**Table 1:** Donor self-reported information and pandemic-specific antibody and CD8 T cell responses in the Toronto cohorts.

			No. donors (n = 105)	Mean age	Received pandemic monovalent vaccine	Detectable pandemic H1N1 titers autumn 2009^a^	Detectable pandemic H1N1 titers summer 2010	CD8 T cell response detected
	All donors	All	68^b^	50	41^c^ (61)	10 (15)	23 (34)	44 (65)
Seroprevalence cohort		Vaccinated	41	54	41 (100)	4 (10)	19 (46)	25 (61)
		Unvaccinated	26	43	0 (0)	6 (23)	4 (15)	18 (69)
		All	20	44	5 (25)	NA	15 (75)	10 (50)
	Confirmed cases	Vaccinated	5	55	5 (100)	NA	5 (100)	3 (60)
Case/control cohort		Unvaccinated	15	40	0 (0)	NA	10 (67)	7 (47)
		All	17	51	9 (53)	NA	4 (24)	7 (41)
	Controls	Vaccinated	9	50	9 (100)	NA	3 (33)	4 (44)
		Unvaccinated	8	52	0 (0)	NA	1 (13)	3 (38)

a a titer of 40 was used as the cutoff for seropositivity.

b one donor did not provide vaccination history and was therefore not included in the vaccination stratification and subsequent vaccine recipient frequency.

c number of donors within category; parentheses indicates percentage.

### Frequency and functional analysis of T cell memory to influenza in the Toronto cohort

**Figure 2:**
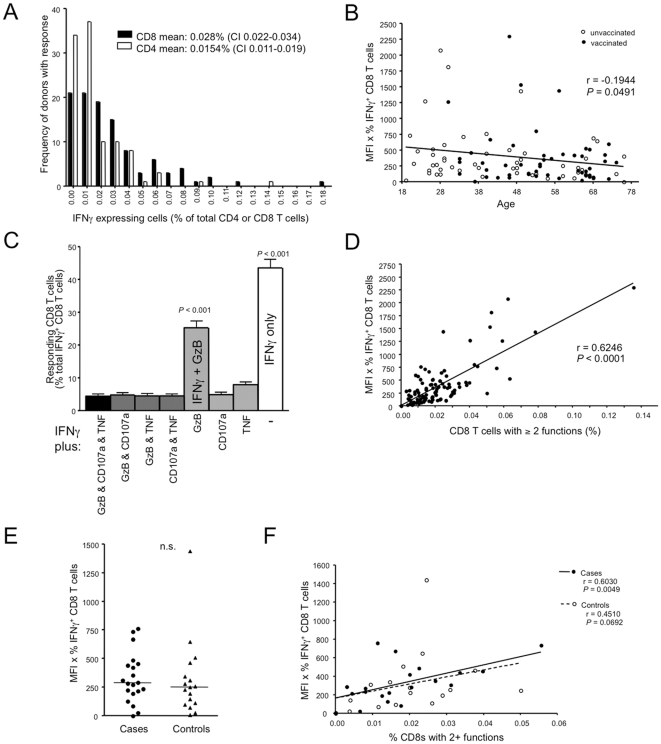
T cell analysis in the Toronto seroprevalence and case/control cohorts. (A) Bin separation of IFNγ responses in CD8 and CD4 T cells specific to pH1N1 stimulation. Frequencies have been corrected for background IFNγ production in LCMV and unstimulated control cultures. (B) Spearman correlation between pH1N1-responding CD8 T cells and donor age. (C) Combinations of effector molecule expression of IFNγ^+^ CD8 T cells from the responder subset. P values above the bars indicate the level of statistical significance compared to all other bars as determined by ANOVA and Tukey test. (D) Spearman correlation between the CD8 T cell response to pH1N1 and the frequency of responding cells with multiple effector functions. (E) CD8 T cell response in case and control subjects. Groups were compared using a nonparametic Mann-Whitney test. (F) Spearman correlation for pH1N1 response and frequency of CD8 T cells with multiple effector functions in cases and controls.

Based on the above analysis, we found detectable influenza-specific CD8 T cell responses (IFNγ positive cells) in over half of all donors, with 18% of subjects tested showing responses greater than 0.05% (Figure 2A). The mean percentage of IFNγ^+^ CD8 T cells, after background correction, was 0.038% in responders, compared to a mean of 0.015% in non-responders (medians 0.028% and 0.006% respectively) (Figure 2A). Detectable CD4 T cell responses were generally much lower than those of CD8 T cells and showed higher background (mean CD4 IFNγ^+^  =  0.015% after background correction). The level of the CD8 response declined slightly with age (Figure 2B).

In addition to measuring IFNγ, the major cytokine produced upon restimulation of virus-specific CD8 T cells, we analyzed cells for the simultaneous production of TNF as well as for expression of granzyme B (required for killing of virus-infected cells) and CD107a (a marker of degranulation) for donors with detectable T cell responses. Almost half of influenza-specific CD8 T cells produced only IFNγ However, a significant proportion of the T cells also made granzyme B (Figure 2C) and there were also small but detectable populations of T cells that produced other combinations of effector molecules. In other viral infections, the presence of multifunctional CD4/CD8 T cells correlates with viral control [20], [21]. Moreover, these multifunctional T cells have been reported to produce larger amounts of cytokines per cell [22]. This was also the case for the memory T cells specific for influenza in our study, as donors with the highest CD8 recall response (MFI x frequency) were those with the most multifunctional T cells (Figure 2D). The prevalence of multifunctional T cells did not appreciably change as a function of donor age (data not shown).

### Cases and controls have an indistinguishable frequency of influenza-specific memory CD8 T cells post-pandemic

A PCR test for pH1N1 from nasopharyngeal swabs was performed on all case/control participants. In this cohort, we found no significant difference between cases and controls for the CD8 T cell responses to pH1N1 in PBMC samples collected 8–10 months after PCR testing (Figure 2E). As with the total T cell responder population, both PCR-confirmed cases and controls with increased IFNγ^+^ CD8 T cell populations had a higher frequency of responding cells with multiple effector functions, although no observable difference was detected between the proportions of multifunctional T cells in cases versus controls (Figure 2F).

### The T cell response against pH1N1 is greatly enhanced after the onset of PCR-confirmed pH1N1 illness and gradually returns to pre-pandemic baseline

**Figure 3:**
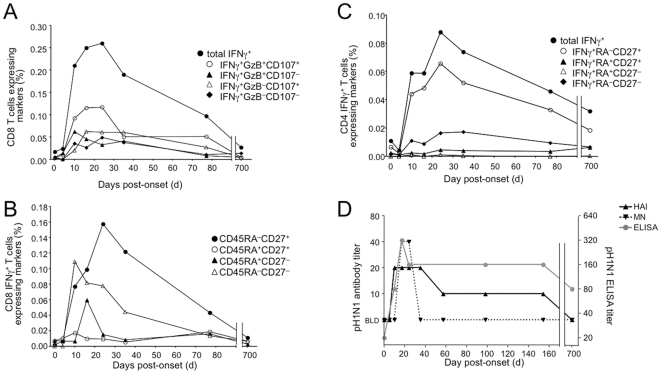
Acute and persisting antibody and memory T cell responses to pandemic H1N1 infection in one PCR case-confirmed donor. Longitudinal samples of unfractionated PBMC were challenged with influenza virus or controls for 18h. (A) Frequency of pandemic H1N1-responsive CD8 T cells out of total CD8 T cells as measured by IFNγ staining. IFNγ responsive CD8 T cells were also sub-divided by expression of other effector markers, granzyme B and CD107a. (B) Memory phenotypes of influenza-responsive CD8 T cells at various times post-onset of influenza symptoms. (C) Frequency and phenotypes of IFNγ^+^ CD4 T cells after pandemic H1N1 challenge. (D) Antibody titers in serum as detected by microneutralization (MN), hemagglutination inhibition (HAI), and a pandemic H1-specific ELISA assay. BLD  =  below the limits of detection.

As T cell responses were measured approximately 8–10 months post-pandemic, it was not possible from the cohort analysis to know how large the response might have been shortly after exposure. However, the availability of longitudinal PBMC samples from one PCR-confirmed pandemic H1N1 case, before and after infection, offered the opportunity to follow an acute T cell response to influenza infection in a human subject. Prior to infection, this donor had a weak positive CD8 T cell response to pH1N1 (Figure 3A). By 10 days post-onset of illness, there was a dramatic increase in IFNγ-producing CD8 T cells. The majority of these cells co-expressed granzyme B as well as CD107, a phenotype associated with T cells' ability to kill virus-infected cells. The peak CD8 T cell response was attained approximately 3 weeks after the initial onset of illness, with a modest decline observed by day 78. Cells expressing all 3 functional markers were maintained and the total IFNγ^+^ CD8 T cell response remained above the baseline measurement even two and a half months post-infection (Figure 3A). However, by day 700, the frequency of CD8 T cells responding to pH1N1 had returned to pre-infection baseline frequencies and was at a level similar to that of the cross sectional cohort at one year post-pandemic. At these later time points, multifunctional T cells were largely absent in both the cohort and the longitudinal donor.

In the longitudinal samples, we observed differential kinetics of expansion for influenza-specific CD8 T cell memory responses delineated on the basis of their expression of two surface markers, CD45RA and CD27 (Figure 3B). CD27 is a costimulatory receptor on T cells that is lost from effector memory cells in some viral infections and whose loss is often associated with a more differentiated effector cell phenotype [23]–[25]. CD45RA is a marker that is normally present on naïve T cells, disappears upon their differentiation to effector and memory T cells, but can reappear on more terminally differentiated effector cells [23]–[27]. The CD45RA^−^CD27^−^ influenza-specific T cell population was the first response to peak, and based on the absence of CD27, likely represented reactivation of a pre-existing effector/memory population. A population of effector-like T cells (CD45RA^+^CD27^−^) transiently increased before the final, largest wave of influenza-specific T cells emerged. The last wave of influenza-specific T cells expressed CD27, suggesting expansion from a less differentiated memory T cell pool. These CD45RA^−^CD27^+^ CD8 T cells were the most persistent phenotype in the longitudinal donor, similar to those detected in the Toronto cohort post-pandemic (data not shown). We also observed three distinct influenza-specific CD4 T cell subsets, although their kinetics of expansion were not distinguishable (Figure 3C).

We tested sera from the longitudinal donor and found that pandemic influenza-specific antibodies were low, except for weakly positive titers detected by MN and ELISA (but not HAI) between 2–3 weeks post-infection (Figure 3D). Two subsequent vaccinations containing pH1N1 antigen did not result in long-term persisting protective titers against pH1N1.

### Similar CD8 recall T cell responses to H1N1 from 1934 and 2009 implies cross-reactive T cell responses

**Figure 4:**
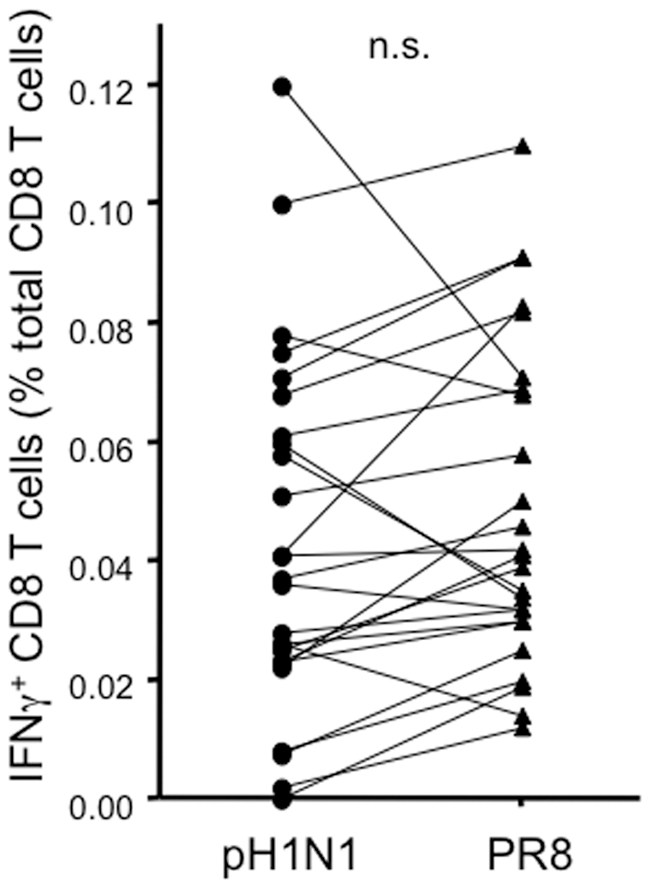
CD8 T cell IFNγ responses to pH1N1 may be cross-reactive with other influenza strains. IFNγ response to A/California/7/2009 and A/PR8 in a set of 25 donors. A paired t test was used to compare the differentially stimulated cultures on a per-donor basis.

CD8 T cell epitopes are mainly derived from internal viral proteins and are from the most conserved part of the influenza virus. Indeed the sequences of influenza NP and M proteins have changed little between the 1918 and 2009 pandemic strains and T cells specific for A/California 2009 are cross-reactive with the 1918 H1N1 pandemic strain [10]. Therefore, it was likely that a significant proportion of the T cell responses we observed to A/California 2009 would cross-react with other H1N1 strains. To test this hypothesis we compared the responses of a subset of donors to A/California 2009 and a 1934 virus, A/PR8/34. In the 25 donors tested with the A/PR8/34 virus, the proportion of influenza-responding CD8 T cells was indistinguishable from the pH1N1 strain (Figure 4), consistent with the possibility that a significant proportion of the CD8 T cells that respond to A/California 2009 are cross-reactive.

### Vaccination after infection with pH1N1 results in higher persisting antibody titers but not T cell responses

**Figure 5:**
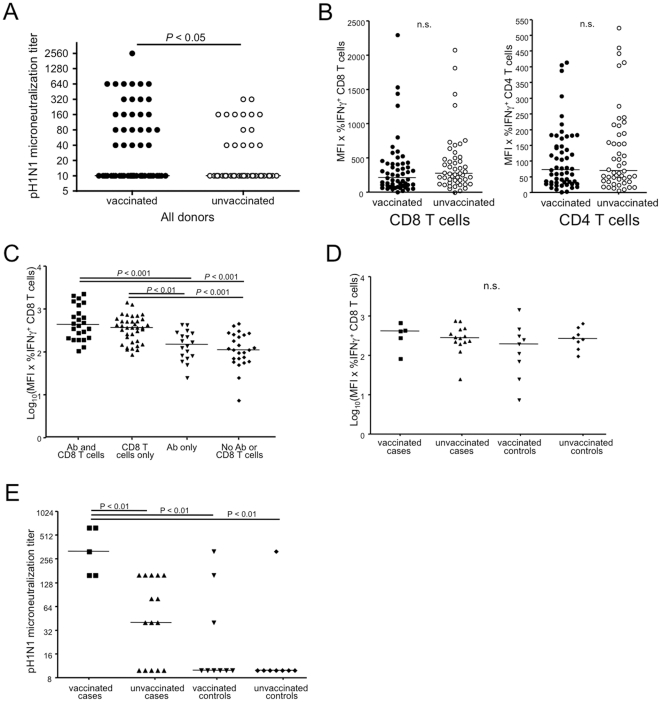
Infection followed by vaccination boosts antibody but not T cell responses to pandemic H1N1. (A) Antibody titers against pH1N1 for vaccinated and unvaccinated donors in the entire cohort 8-10 months post-pandemic. Vaccinations were self-reported from October 2009 to January 2010. A non-parametric Mann-Whitney test was used for statistical significance. (B) CD8 and CD4 responses to pH1N1 for vaccinated and unvaccinated donors in the total Toronto cohort, measured 8-10 months post-pandemic. Groups were compared using a Mann-Whitney test. (C) IFNγ^+^ CD8 T cell responses in donors with both antibody and CD8 T cell responses, T cell responses only, antibodies only, or no antibody or T cell response to pH1N1. Data has been normalized using log transformation to represent Gaussian distribution; groups were compared using ANOVA and Tukey test. (D) Normalized CD8 T cell response in cases and controls with differing vaccination history for pH1N1. Groups were compared by ANOVA and Tukey test. PCR-confirmed infections were reported from April-November 2009; vaccination was self-reported from October 2009-January 2010. (E) Pandemic-specific antibody responses as measured by microneutralization in the case/control cohort, separated by self-reported vaccination history for the monovalent pH1N1 vaccine. PCR-confirmed infections were reported from April-November 2009; vaccination was self-reported from October 2009-January 2010. Nonparametric Kruskal-Wallis and Mann-Whitney tests were performed to determine statistical significance.

Antibody responses to pH1N1 in the seroprevalence and the case/control cohort are summarized in Table 1. Interestingly, as previously noted [16] among unvaccinated but laboratory confirmed infected donors, one third were seronegative for H1N1 A/California/7/2009 by the summer of 2010 as measured by MN (Table 1), HAI, and ELISA (data not shown). However, with the exception of the longitudinal donor, all of the donors with a confirmed infection who were also vaccinated were seropositive for influenza A H1N1 2009 antibodies by summer of 2010 (Table 1). For the seroprevalence cohort, 10% of vaccinated donors were seropositive (≥1∶40) for pH1N1 in the autumn of 2009 (prior to vaccination), increasing to 46% in the summer of 2010, as measured using the microneutralization assay. Unvaccinated donors showed a decline in seropositive titers with time, from 23% in autumn 2009 to 15% by summer 2010. Indeed, vaccinated individuals were more likely to have detectable persisting antibody titers to pH1N1 8-10 months post-pandemic compared to unvaccinated individuals within the entire Toronto cohort (Figure 5A). In contrast to antibody responses, T cell responses were no higher in vaccinated compared to unvaccinated individuals (Figure 5B).

We observed a rather diverse range of antibody titers to pH1N1 in the post-pandemic samples. Indeed, rather surprisingly, it appears possible to have confirmed infection without seroconversion, albeit based on a single longitudinal donor. Thus we wanted to know if the antibody titers reflected the overall level of immune response. In other words, would those with a detectable antibody response be those with the highest T cell response as well. However, we found that the overall level of T cell response was not different between those with an antibody response and those without. Of all donors, 23% had detectable pandemic-specific antibodies as well as influenza-specific CD8 T cells, 35% had only the H1N1-specific CD8 T cells, 17% only antibodies, and 25% had neither detectable antibodies nor pH1N1-specific CD8 T cells (Figure 5C). Those donors who had both pH1N1-reactive T cells and antibodies did not on average have higher T cell responses than those who lacked antibody responses (Figure 5C).

A wide variety of antibody titers were detected even within the cases of the Toronto case/control cohort. Therefore we divided PCR-confirmed cases or controls based on their pH1N1 vaccination status and examined their T cell responses and antibody titers. Interestingly, the CD8 T cell responses in the case/control vaccinated or unvaccinated groups 8-10 months post-pandemic did not vary significantly (Figure 5D), suggesting that CD8 T cell responses in infected persons have returned to baseline levels. However, we found that subjects who were both infected and vaccinated had significantly higher antibody titers than any other group of the case/control cohort, although some naturally infected, unvaccinated donors did have measurable levels of pandemic-specific antibodies (Figure 5E). However, most of the unexposed control donors, regardless of their vaccination status, did not show detectable pH1N1 titers. Taken together, these data show that vaccination within a short time after confirmed infection increases the level of antibody titers observed at 1 year post-pandemic without affecting the level of persisting T cell memory.

## Discussion

In this study we evaluated antibody and T cell memory responses to H1N1 influenza approximately one year post-pandemic. The results show that 61 of 105 donors (58%) had detectable circulating CD8 T cell memory to H1N1 influenza. The magnitude of the T cell memory pool and the markers of T cell effector function measured at 1 year post-pandemic did not differ between cases and controls and was not significantly altered by pH1N1 vaccination. Clinical studies of the adjuvanted pandemic H1N1 vaccine showed very good efficacy for both working age and older adults [28], however these studies were limited to the short term. In our study, which examined antibody titers to pH1N1 8-10 months after vaccination, we found only modest increases in titers in vaccinated compared to unvaccinated donors in the combined cohorts (Fig. 5A). In members of a seroprevalence cohort, there was an increase in seroconversion in vaccinated individuals between 2009 and 2010; however, only 46% of vaccinated donors were seropositive almost one year post-vaccination. Only 75% of confirmed cases in the case-control study were seropositive for pH1N1 in the summer of 2010. However, with the exception of the longitudinal donor, the infected donors who were subsequently vaccinated were all seropositive and had the highest antibody titers 1 year post-pandemic (Table 1 and Fig. 5E). These findings argue that although the serum antibody response to influenza infection or vaccination may be short-lived, there could be value in vaccinating individuals who have had confirmed influenza as this appears to sustain their antibody levels to titers considered protective [29]–[31].

This study offered the rare opportunity to observe the T cell response to influenza virus in real time for one donor. This donor was 55, and therefore it was likely not his first influenza exposure, but rather a recall response. Although interpretation of these results must be tempered, as only one donor was available for longitudinal analysis, this donor was similar in age and had a similar T cell memory pool more than 1 year post-infection to that of the cross-sectional cohort, making it less likely that his response is abnormal. A barely detectable population of influenza-responsive T cells at the pre-infection time point was able to expand more than ten-fold over the course of illness. Although CD8 T cell responses remained high in the blood at least a month after onset of illness, a significant decrease in the responding cells occurred between one and three months after infection. Interestingly, even at somewhat later time points, the frequency of IFNγ-producing CD8 T cells had not yet returned to baseline levels. However, after nearly two years and two subsequent vaccinations, CD8 T cell memory to pH1N1 detected in the blood was very similar to pre-infection levels. This and the fact that most IFNγ CD8 T cell responses to pH1N1 were under 0.1% in the Toronto cohort suggests that influenza-responsive CD8 T cell boosting from influenza infection is transient. Although we do not know the magnitude of the initial response of the cross-sectional cases to influenza infection, based on the analysis of the longitudinal donor, we can speculate that even when present at these low levels, influenza-specific memory T cells can rapidly expand and become polyfunctional post-infection. Thus, although the rate of expansion of these memory T cells may be too slow to control initial infection, CD8 T cells may help prevent serious complications and shorten the duration of symptoms.

The T cells that responded most rapidly to acute influenza infection in the longitudinal donor likely come from pre-existing influenza-specific memory pools. Indeed, the CD8 T cell epitopes to influenza virus are much more conserved than antibody epitopes and have changed little between 1918 and the present day [10], [32]. Consistent with this observation, the magnitude of the memory CD8 T cell response to A/California/2009 and A/PR8/34 was similar regardless of donors' documented history of recent infection and serological status, arguing that a high proportion of the memory pool may be cross-reactive. Some of these cross-reactive T cells may be from pre-existing memory T cells (Fig. 3A), whereas for recently infected donors, some may reflect the recruitment of new T cells into the response

As the inactivated influenza vaccine does not directly increase CD8 T cell responses to influenza [33], concerns have been raised that vaccination may decrease subsequent protection to influenza by preventing the CD8 T cell boosting that comes from periodic natural infection [34]. However, in this study we found that the pool of influenza-reactive memory T cells persisting at 8–10 months post-vaccination or infection did not differ between the two groups. Thus, while vaccination of older people does not increase the CD8 T cell memory pool, neither does it diminish T cell memory relative to natural infection. However, this issue remains relevant in younger people whose primary exposure to influenza antigens may be through vaccination rather than natural infection and who may not develop influenza-specific memory T cell responses.

A limitation of our study is that we measured antibody levels in blood, rather than at the site of neutralization, the upper airways. Moreover, we cannot rule out that memory B cells would give rise to an antibody response at those sites. Notwithstanding this limitation, serum Ig levels are the standard of measurement for the presence of neutralizing antibodies to influenza infection, and both nasal and serum antibodies correlate with protection from influenza-induced disease severity [35]. Epidemiological studies have also shown that influenza-specific antibodies in the blood correlate with protection, and that a 1∶40 titer is the point at which 50% of individuals in a population would be protected [29]–[31].

At one year post-pandemic, the serum antibody levels in a substantial minority of vaccinated or infected individuals were below levels considered seroprotective (<1∶40). This argues that either they did not seroconvert initially, which seems unlikely based on reported seroconversion rates in clinical trials [36], or did not maintain antibody levels. This is contrary to antibody responses generated from many other vaccines and infections. For example, smallpox-specific antibodies continue to be detectable in serum over 60 years after vaccination [37].

Despite the low levels of circulating serum antibody detected at 1 year post-influenza infection, recent studies have shown rapid mobilization of memory B cells as demonstrated by the isolation of B cell plasmablasts that produce influenza-specific antibodies at day 5 post-infection [7], [38]. The authors suggest that this rapid mobilization may allow memory B cells to increase antibody levels rapidly enough to provide some protection in donors with appropriate memory B cells to influenza [38]. In the absence of measurement of B cell memory populations or of antibody titers in the first few weeks post-infection or vaccination, we cannot state whether the substantial minority of subjects who failed to maintain titers to H1N1 above 1∶40 at 1 year post-vaccination or infection were in fact capable of mounting this response transiently. However, in following a single donor post-H1N1 infection, we observed only a small increase in neutralizing antibody responses to pandemic infection as detected by microneutralization assay and ELISA (but undetectable by HAI). This suggests a rather poor antibody response to pH1N1 infection in this individual. Moreover, this weak antibody response had returned to baseline pre-infection levels by 700 days, despite two intervening H1N1 vaccinations. We speculate that the poor antibody response from this single longitudinal donor could be due to an exhaustion of his memory B cell population for this influenza strain, perhaps due to repetitive exposure to influenza over a lifetime. Further studies will be required to compare both serum antibody and memory B cell responses during the acute phase of influenza infection to determine the source of the weak antibody response to pH1N1.

In sum, this study shows that almost 60% of a Toronto cohort had cross-reactive memory T cell responses to influenza virus at one year post-pandemic. The size of the long-lived pH1N1-reactive memory T cell pool is not different between infected, vaccinated and unvaccinated individuals, consistent with the finding that the memory T cell response to influenza increases only transiently post-infection and is not boosted by current vaccines. However, based on one donor, we find that these T cells can expand significantly post-infection. While too late to prevent infection, these cross-reactive T cells may offer some contribution to viral control and improved outcome. There appear to be relatively low levels of antibody to influenza persisting at 1 year post-infection or vaccination, suggesting that serum antibody levels are not maintained at high levels post vaccination or infection. However, the finding that vaccination can at least transiently increase antibody levels seen at one year post-pandemic in individuals who had confirmed infections argues that even in those recently infected with influenza, taking the subsequent seasonal vaccine may be able to increase their level of antibody to titers that have been shown on a population basis to be protective.
